# A Genetic Screen Based on *in Vivo* RNA Imaging Reveals Centrosome-Independent Mechanisms for Localizing *gurken* Transcripts in *Drosophila*

**DOI:** 10.1534/g3.114.010462

**Published:** 2014-02-14

**Authors:** Rippei Hayashi, S. Mark Wainwright, Sophie J. Liddell, Sheena M. Pinchin, Stuart Horswell, David Ish-Horowicz

**Affiliations:** * Developmental Genetics Laboratory and; † Bioinformatics Unit, London Research Institute, 44 Lincoln's Inn Fields, London WC2A 3LY, UK

**Keywords:** oogenesis, RNA localization, microtubules, whole-genome sequencing, mutant mapping

## Abstract

We have screened chromosome arm 3L for ethyl methanesulfonate−induced mutations that disrupt localization of fluorescently labeled *gurken* (*grk*) messenger (m)RNA, whose transport along microtubules establishes both major body axes of the developing *Drosophila* oocyte. Rapid identification of causative mutations by single-nucleotide polymorphism recombinational mapping and whole-genomic sequencing allowed us to define nine complementation groups affecting *grk* mRNA localization and other aspects of oogenesis, including alleles of *elg1*, *scaf6*, *quemao*, *nudE*, *Tsc2/gigas*, *rasp*, and *Chd5/Wrb*, and several null alleles of the *armitage* Piwi-pathway gene. Analysis of a newly induced *kinesin light chain* allele shows that kinesin motor activity is required for both efficient *grk* mRNA localization and oocyte centrosome integrity. We also show that initiation of the dorsoanterior localization of *grk* mRNA precedes centrosome localization, suggesting that microtubule self-organization contributes to breaking axial symmetry to generate a unique dorsoventral axis.

Asymmetric messenger (m)RNA localization in the cytoplasm acts to restrict the sites of protein synthesis, particularly in large or polarized cells (Martin and Ephrussi 2009). For example, specification and maintenance of *Drosophila* oocyte fate depends on the transport of selected mRNAs along microtubules (MTs) from accessory nurse cells to the adjacent and interconnected oocyte (St. Johnston 2005). The minus-end-directed motor dynein and its cofactors Bicaudal-D (BicD) and Egalitarian (Egl) are required for selective RNA transport into the oocyte and for the localization of certain transcripts in later-stage oocytes (Bullock and Ish-Horowicz 2001; MacDougall *et al.* 2003; Navarro *et al.* 2004). Posterior localization of the mRNA encoding the germline determinant Oskar depends on the plus-end-directed motor kinesin-1 (Brendza *et al.* 2000). Genetic and biochemical experiments have shown that the ultimate destinations of transported RNAs depend on recognition of cargo RNAs by appropriate MT motors and on the organizational architecture of the MT cytoskeleton (MacDougall *et al.* 2003; Dienstbier *et al.* 2009; Parton *et al.* 2011).

Dynein-dependent RNA transport in *Drosophila* eggs and oocytes relies on short RNA signals that are presumably recognized by motor components and adapter proteins. However, the basis for the signals’ specificity and recognition is unclear. One such signal forms a novel helical RNA structure (Bullock *et al.* 2010), but its generality in directing RNA transport is not currently known. There is strong *in vitro* evidence that the Egl protein acts as an adapter between dynein and cargo mRNA (Dienstbier *et al.* 2009), but some signals may have different structures and operate via other adapters.

A particularly significant target of dynein-mediated transport is *gurken* (*grk*) whose transcript localization is key to establishing the prospective body axes of the future embryo. *grk* mRNA localizes posteriorly in early oocytes and is translated during stage 5 into a transforming growth factor-α−like protein that signals to overlying, somatic follicle cells to specify their posterior character (Gonzalez-Reyes *et al.* 1995). During this stage, the minus-ends of MTs are orientated predominantly toward the oocyte posterior.

During stages 7−8, *grk* transcripts delocalize to a dorsoanterior corner, allowing localized Grk signaling to establish the dorsoventral axis of the oocyte (Neuman-Silberberg and Schüpbach 1993). At this time, the nucleus and the oocyte centrosome also migrate from the oocyte posterior to its dorsoanterior corner (Januschke *et al.* 2006), and the cytoskeleton is remodeled so that MTs with anteriorly orientated minus-ends predominate (Theurkauf *et al.* 1992). How MTs are reorganized at this stage remains controversial, but a recent study has suggested that anterior migration of the oocyte nucleus during stage 7 is due to its being pushed by the posterior-lying centrosome (Zhao *et al.* 2012).

Several studies indicate that MTs can nucleate from the lateral and anterior cortex of the oocyte and from the centrosome and the nuclear envelope (Cha *et al.* 2002; Januschke *et al.* 2006; Parton *et al.* 2011). It is unclear whether the nucleus and the centrosome localize first or whether cortical MTs prefigure organelle localization, nor is it understood how different classes of MTs might contribute to the asymmetric localization of *grk* mRNA.

In this paper, we report a novel genetic screen for maternal factors needed to localize fluorescently labeled endogenous *grk* transcripts during oogenesis. We also describe the combined use of whole-genome sequencing (WGS) and single-nucleotide polymorphism (SNP)-marked recombination to rapidly identify new genes required for *grk* localization, egg-chamber morphogenesis, and correct organization of the MT cytoskeleton. Finally, we present novel analysis of wild-type and *kinesin light chain* (*klc*) mutant oocytes that reveals roles for centrosome-dependent and -independent MTs in *grk* mRNA localization and axial patterning.

## Materials and Methods

### Genetic screen

**Figure 1:**
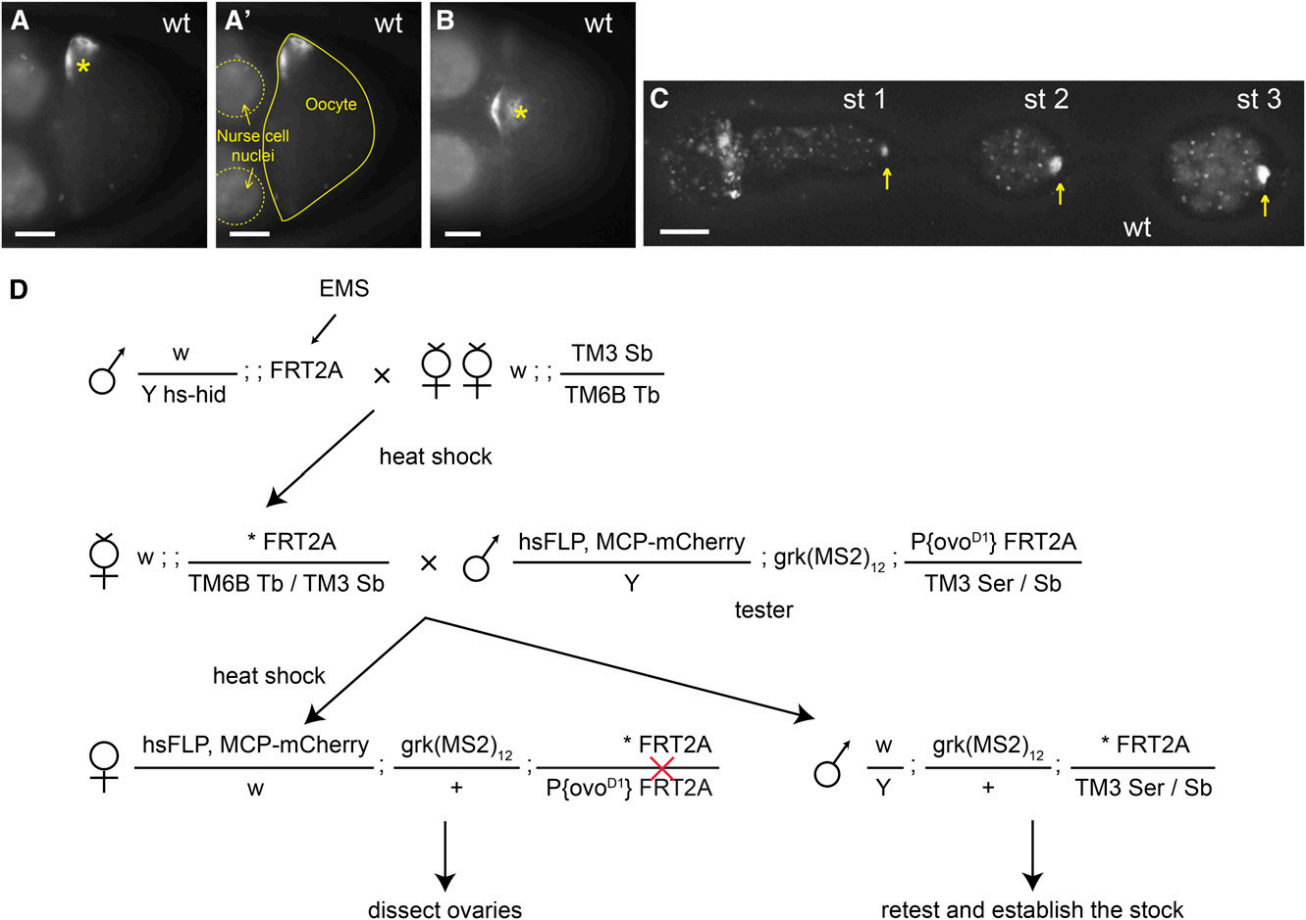
Genetic screen based on *in vivo* RNA imaging. (A−C) *grk* transcript localization in wild-type egg-chambers. (A, B) Localization of grk*mCherry (MCP-labeled grk transcripts) as visualized in differently orientated stage 8−9 wild-type oocytes: the nucleus (asterisk) is at the side or at the top center of the anterior end in A and B, respectively. (A′) Same image as in (A) outlining the oocyte (solid line) and two nurse cell nuclei (dashed lines). (C) grk*mCherry in the germarium and stage 1−3 egg-chambers. grk*mCherry localizes to the wild-type oocyte, which is at the posterior of the egg-chambers (yellow arrows). (D) Crossing scheme for generating mosaic females with homozygous mutant germline. Asterisk indicates the mutagenized chromosome. *hs-hid* males are selectively eliminated by heat shock during larval stages. The red cross indicates a FLP-induced mitotic recombination event between the two *FRT* sequences. All developing egg-chambers are homozygous for induced mutations on 3L, because they lack the dominant female-sterile *ovo^D1^* gene. Scale bars = 20 μm.

Details of fly stocks, mutagenesis, and the screen are described in Figure 1 and the Supporting Information, File S1. In summary, novel mutations were identified by dissecting one to three females of the genotype *hsFLP*, *nanos*::*MCP-mCherry/w*; *grk(MS2)_12_/w*; *FRT2A/ P{ovo^D1^} FRT2A (“*FRT2A” representing the mutagenized chromosome 3L) 8−10 d after heat-shock to induce homozygous mutant germlines and stage 8−9 oocytes screened for the distribution of fluorescently marked *grk* mRNA (grk*mCherry). Only germline cysts that are homozygous for the mutagenized chromosome lack the dominant female-sterile mutation *ovo^D1^* and can develop to later stages.

Approximately one-third of the mutagenized lines lacked egg-chambers that reached stage 8−9, perhaps reflecting mutation that cause early defects in oogenesis. To detect such mutations, ovaries larger than those of *ovo^D1^* flies were scored for the presence and position of the grk*mCherry spot that marks the early oocyte (Figure 1C).

### WGS and SNP mapping

Parental and *ru h th st cu st e ca* (*rucuca*) chromosomes were sequenced at 168- and 28-fold coverage, respectively. These levels of coverage allowed us to reassemble ~99% of the euchromatic coding genome for each stock and provided a high density of euchromatic SNP markers (averaging 1 per 1.5 kb) for recombination mapping. Individual mutant chromosomes were sequenced at about 20-fold coverage.

**Figure 2:**
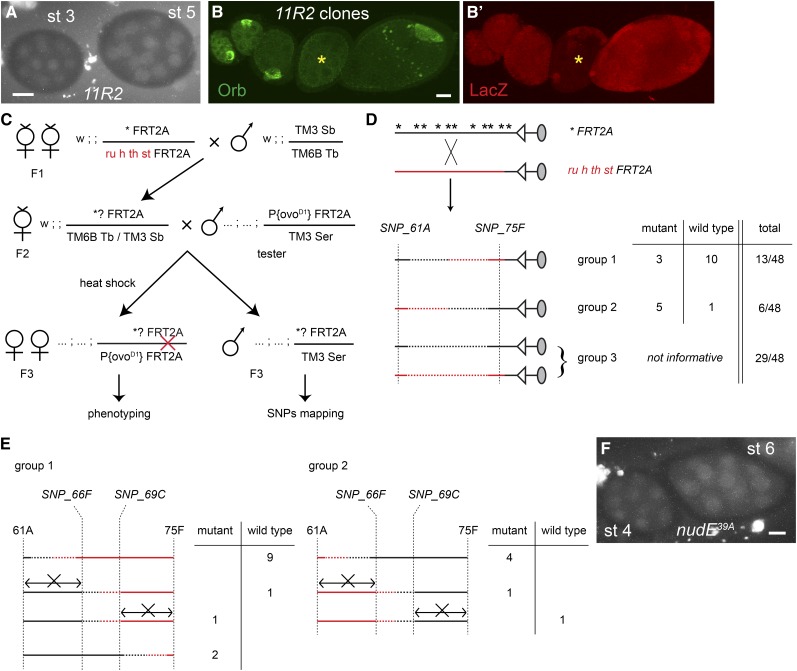
*11R2* is an allele of *nudE*: overview of the single-nucleotide polymorphism (SNP) mapping. (A and F) grk*mCherry in *11R2* (A) and *nudE^39A^* (F) stage 3−6 egg chambers. Lack of localized grk*mCherry indicates the absence of oocyte. (B) Lack of Orb staining in a *11R2* germline clone (*, marked by the absence of LacZ expression; B′) confirms the failure to form an oocyte. Shown is a projection of a series of z-stack confocal sections that cover the whole egg chamber. (C) Crossing scheme for the SNP mapping. Individual F2 females are mated to the tester males (as in the primary screen), and F3 progeny are used for phenotyping (in germline clones) and genotyping. (D, E) summary of SNP genotyping. (D) 48 putative recombinants were genotyped for the SNPs at 61A and at 75F. The ratio of mutant and wild-type recombinants in each group yields an approximate map position. Nonrecombinant lines (group 3) are excluded from the subsequent assays. (E) Genotyping by exclusion shows that the causative lesion lies between 66F and 69C. If necessary, further mapping could have been performed using the four remaining recombinants. Scale bars = 20 μm.

The basis for SNP-based mapping of causative mutations is presented in Figure 2. Recombinant females (*?FRT2A/TM3, *Sb or TM6*, *Tb* F2 virgin recombinant females; “*?” representing the possibly mutant recombinant chromosome) were individually mated with tester males carrying *TM3*, *Ser* for phenotypic retesting, SNP mapping, and to make stocks of informative recombinants. DNA was extracted from individual *?FRT2A/TM3, *Ser* F3 males and used for high-throughput allelic-discrimination polymerase chain reaction (KASPAr; LGC Genomics, http://www.lgcgenomics.com/), which provide a two-color fluorescence assay for SNP genotyping in microtiter plates (File S2). F3 females showing recombination between proximal and distal SNPs were tested phenotypically as in the primary screen. Additional details are presented in the section *Results* and File S1.

### Other methods

Plasmid construction, RNA quantification, antibody staining, *in situ* hybridization, and X-gal staining were performed as described in File S1.

## Results

### Genetic screen based on sensitive *in vivo* fluorescent labeling of *grk* mRNA

*grk* transcripts have previously been visualized *in vivo* by labeling with a fluorescent MS2 Coat Protein (MCP) fusion protein, which binds to endogenous *grk* transcripts that include 12 repeats of the MCP binding-site (*grk-(MS2)_12_*; Jaramillo *et al.* 2008). To sensitize the system for large-scale screening, we used MCP fused to the more photostable mCherry (Shaner *et al.* 2004) and excluded fluorescence from the follicle cells by driving MCP-mCherry expression selectively in the germline using the *nanos* promoter and a shortened 3′UTR from *fs(1)K10* that both stabilizes germline transcripts and restricts them to the nurse cells (Serano *et al.* 1994).

Together, these changes allow rapid and sensitive visualization of the tagged *grk* transcript (which we refer to as grk*mCherry) in live ovaries. grk*mCherry and endogenous *grk* transcripts both localize tightly around the nucleus at the dorsoanterior corner of the oocyte from stage 8 and appear as a dorsoanterior crescent or perinuclear halo depending on whether viewed from the top or side, respectively (Figure 1, A, A′, and B). In younger oocytes, grk*mCherry shows a distinctive spot at the posterior of the early egg-chamber due to transport of *grk* mRNA into the developing oocyte (Neuman-Silberberg and Schüpbach 1993).

We used ethyl methanesulfonate (EMS) mutagenesis and the FLP-*ovo^D1^*-dominant-female-sterile system to generate germline clones of newly- induced mutations on chromosome arm 3L and screened for those affecting *grk* mRNA localization (Figure 1D; *Materials and Methods*; Chou *et al.* 1993). We scored 4911 independently mutagenized lines and found 39 lines with apparent defects in grk*mCherry localization, 11 of whose phenotypes repeated on rescreening. We also identified several mutations affecting egg-chamber and oocyte morphology.

### Identification of newly induced mutations by WGS and SNP-based recombinational mapping

A standard EMS dose in *Drosophila melanogaster* (25 mM) induces an average of one mutation per 400 kb (Cooper *et al.* 2008), corresponding to about 10 mutations per chromosome arm that affect protein-coding regions (Misra *et al.* 2002). These were easily identified by deep genomic sequencing of the parental and mutated chromosomes (*Materials and Methods*; File S1). To determine which mutation was causative, we first tested for allelism. If present, we compared the genome sequences of two alleles to identify a gene on 3L that is mutated in each allele. For mutants represented by single alleles, we used high-throughput SNP markers and genetic recombination to map the mutation to a 2- to 3-Mb region (~10% of a chromosome arm), which is usually sufficient to restrict the number of candidate causative coding mutations to one or two. The phenotype and the molecular lesions of all mutants are summarized in Table 1, Table 2, and Table 3.

**Table 1:** Mutants identified from the screen

	gene	alleles	*grk* mRNA localization in stage 6	*grk* mRNA localization in stage 9	other phenotypes
mislocalised *grk* mRNA in late stages	*armitage* (*armi*)	*3R13*, *9R1*, *10M12*, *11R3*, *11S9*, *13P2*, *13P5*, *14P4*	diffuses internally	weakly accumulates around the nucleus, spreads along anterior periphery	defective follicle cell development
	saturn*: kinesin light chain (*klc*) *& maelstrom* (*mael*)	*5R12*	diffuses internally	no longer localizes to the anterior periphery nor to the nucleus	mispositioned nucleus
	*scaf6* + unmapped mutation	*12M9*	diffuses internally	weakly accumulates around the nucleus, spreads along anterior periphery	defective reproduction of germline stem cells
	*elg1*	*3R7*	localizes posteriorly as in wild type	spreads along anterior periphery	defective nurse cell DNA endoreplication

			Phenotypes		
other mutants	*nudE*	*11R2*	Oocyte is misspecified. Most egg chambers contain 16 nurse cells.		
	*quemao* (*qm*)	*5R4*, *11S5*	Plasma membranes of nurse cells and the oocyte collapse. Released ring canals cluster. *grk* mRNA colocalizes to the ring canal cluster.		
	*Tsc2/gigas*	*1R8*, *2R5*, *11R10*	Oocyte is frequently mispositioned.		
	*rasp*	*11R5*, *13P2*, *13P10*, *13P13*	Mature eggs have fused appendages. Gurken proteins diffuse in the cytoplasm.		
	*Chd5/Wrb*	*9R9*, *13P2*	Disorganized nurse cells in late stage 10.		

* *saturn* (*5R12*) is a synthetic phenotype caused by mutations in *klc* and *mael* (see Results).

**Table 2:** Molecular lesions identified by whole-genome sequencing

Allele	Affected Gene(s)	Nucleotide Change	Translational Change
*5R12*	*klc*	AG>GG	Truncation due to mutated intron 2 acceptor
	*mael*	GAG>AAG	E131 > R
*12M9*	*scaf6*	CAG>TAG	Q560 > stop
*3R7*	*elg1*	TGG>TGA	W212 > stop
*11R2*	*nudE*	CAG>TAG	Q33 > stop
*5R4*	*qm*	CAG>TAG	Q116 > stop
*11S5*	*qm*	GCC > GTC	A104 > V
*1R8*	*Tsc2/gigas*	AG>AA	Truncation due to mutated intron 11 acceptor
*2R5*	*Tsc2/gigas*	GTG > GAG	V1726 > E
*9R9*	*Chd/Wrb*	ATAaccgtgtttaTCA > ATA—TCA	Truncation after 107 aa due to 10 bp deletion
*13P2*	*Chd/Wrb*	TGG>TGA	W158 > stop
*11R5*	*rasp*	TAT>TAA	Y210 > stop
*13P10*	*rasp*	ATT|gtcacaat|GTC > ATTgtcacaat-HOBO-gtcacaatGTC	Insertion of HOBO transposon at 177 aa

The mutated bases are underlined.

**Table 3:** Molecular lesions of *armitage* alleles

Allele Name	Amino Acid Change	Domain	*grk* Mislocalization	Eggs Laid by Hemizygous Females
*3R13*, *9R1*	Q315 > stop	C-terminal truncation	+	–
*11R3*	G583 > D	Unknown domain	+	–
*14P4*	Q674 > stop	C-terminal truncation	+	–
*13P2*	G728 > E	Helicase domain	+	–
*10M12*	Splicing donor of intron 7 (GT to AT)	Exon 8 skipped, which truncates after aa 1082	+	–
*11S9*	E1082 > K	Helicase domain	+	+
*13P5*	R1121 > stop	C-terminus of helicase domain truncated	+	–

The helicase domain lies between amino acids 698−1132 of the 1188 amino acids Genbank accession no ABX00729.1. Df(3L)E1 was used to make flies hemizygous for each allele.

To illustrate the strategy, we present the mapping of *11R2*, a mutation that causes oocyte misspecification and that was not allelic to other mutations from our screen. Germline clones of *11R2* lack both the early, oocyte-specific spot of grk*mCherry expression (Figure 2A). They also fail to express another oocyte marker, Orb (Figure 2B), confirming that no oocyte has been specified.

The principle and crosses for the mapping are illustrated in Figure 2. The mutated chromosome *FRT2A was mated with a presequenced *ru h th st FRT2A* chromosome. F2-recombinant females were mated with tester males and, prior to phenotyping, F3 animals were SNP-genotyped using a rapid single-fly polymerase chain reaction assay based on two-color fluorescence that can be assayed in microtiter plates (Figure 1D, Figure 2C, File S1, File S2; and *Materials and Methods*). SNP genotyping was performed in two stages over a single generation: individual F3-recombinant males were identified first by genotyping for SNPs at each end of the chromosome arm (Figure 2D; *Materials and Methods*), allowing early discard of nonrecombinant flies and crosses. Additional SNPs were then used for “digital” fine mapping to define a minimal causative interval by exclusion (Figure 2E). Further technical details are in the section *Materials and Methods* and File S1.

For *11R2*, 19 of 48 F2 females had recombined between tester distal and proximal SNPs (SNP_61A and SNP_75F, respectively in Figure 2D); 3 of 13 recombinants with distal *FRT2A* homology (group 1) and 5 of 6 recombinants with proximal *FRT2A* homology (group 2) were mutant, mapping the mutation closer to 75F than to 61A. Internal SNPs localized the mutation between 66F and 69C, based on a mutant recombinant that excludes the 69C−75F interval and a wild-type recombinant that excludes the region distal to 66F (Figure 2E).

WGS of the original mutant chromosome revealed a nonsense mutation in the region of interest: Q33 > stop in the *nudE* gene (at 67D). NudE is a component of the dynein motor complex (Shu *et al.* 2004; McKenney *et al.* 2010) that is implicated in several transport processes linked to oocyte specification: fusome organization, centrosome migration, and RNA transport into the pro-oocyte (Theurkauf *et al.* 1993; Bolivar *et al.* 2001; Navarro *et al.* 2004). We confirmed that the *nudE* nonsense mutation explains the *11R2* phenotype by showing that germline clones of *nudE^39A^*, a previously described null allele (Wainman *et al.* 2009), also fail to specify an oocyte (Figure 2F).

### Mutations affecting egg-chamber morphology

**Figure 3:**
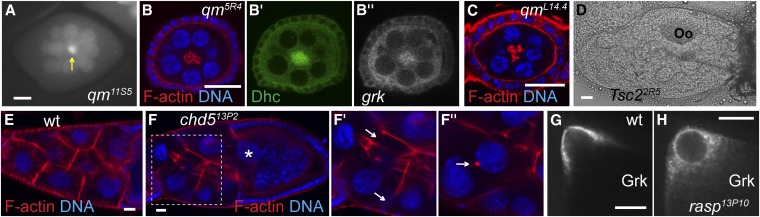
Phenotypes of other mutants identified in the screen. (A) grk*mCherry localizes to the center of the *qm^11S5^* egg-chamber (arrow). (B−B′′) Clustering of ring canals in center of *qm^5R4^* oocytes as revealed by coincident central staining of mutant oocyte for F-actin (phalloidin, B), Dynein heavy chain (Dhc, B′), and grk*mCherry (*grk*, B′′). The lack of F-actin staining between nurse cell nuclei suggests that the cell membranes have collapsed. (C) Staining of F-actin (phalloidin) in *qm^L14.4^* germline clone. (D) Laterally mispositioned oocyte (Oo) in a *Tsc2^2R5^* mutant stage 7−9 egg-chamber. (E, F) Phalloidin staining of wild-type (E) and *Chd5^13P^*2 (F) stage 10B egg-chambers showing discontinuous actin filaments (arrows in F′) and a nurse cell nucleus (asterisk) going beyond the border of nurse cells and the oocyte. A different z-section of the same egg-chamber shows a collapsed ring canal (arrow in F′′). (G, H) Grk protein is more diffuse in the cytoplasm of *rasp^13P10^* (H) than wild-type (G) stage 8−9 egg-chambers. scale bars = 20 μm.

The background cytoplasmic fluorescence of MCP-mCherry allowed us to identify a number of mutations with defects in oocyte positioning or the morphology of egg chamber. Two allelic mutations (*5R4* and *11S5*) are associated with lesions in the *quemao* (*qm*) gene (Table 2) encoding geranylgeranyl pyrophosphate synthase, which synthesizes a lipid substrate for protein prenylation. Most mutant egg-chambers older than stage 5 have unevenly spaced nurse cell nuclei (Figure 3A). They also lack clear boundaries between the nurse cells and the oocyte, suggesting that nurse-cell plasma membranes have collapsed (Figure 3B). The *qm* mutations are indeed responsible for the oocyte phenotypes because germline clones of the previously described null allele (Lai *et al.* 1998) have the same maternal phenotype (Figure 3C).

In *qm* mutant egg-chambers, the ring canals are clustered together centrally (Figure 3, B and C), and Dynein heavy chain (Dhc), which accumulates at MT minus-ends, is enriched adjacent to the ring canal aggregates (Figure 3B′), supporting the idea that ring canals can nucleate nurse cell MTs to facilitate the transcript transport from nurse cells to the oocyte (Clark *et al.* 2007; Mische *et al.* 2007). *grk* mRNA and Dhc still colocalize, indicating that the transcripts are still being transported to MT minus-ends (Figure 3, B′ and B′′).

We also recovered three complementation groups affecting egg-chamber or egg-shell morphology in which *grk* localization appears normal (Table 1). One set of three alleles (*1R8*, *2R5*, and *11R10*) showed frequent oocyte mispositioning (Figure 3D) and mutant egg chambers that deteriorate before stage 10. Sequencing two alleles revealed mutations in *Tsc2*/*gigas* (Table 2), which encodes a negative regulator of cell cycle and tissue growth through the target of rapamycin pathway (Ito and Rubin 1999; Potter *et al.* 2001; Tapon *et al.* 2001). All three alleles fail to complement each other or *Tsc2^192.1^*, a previously described null allele.

Two allelic mutations (*9R9* and *13P2*) affect *CG32022* (Table 2), a gene that encodes a homolog of human Congenital Heart Disease 5 (Chd5)/Tryptophan-rich basic protein (Wrb), which is required for protein insertion into the endoplasmic reticulum membrane (Favaloro *et al.* 2010). The arrangement of nurse cells is disorganized in late stage 10 *Chd5* mutant oocytes, and their nuclei often invade the oocyte (Figure 3, F and F′′). Several aspects of the mutant phenotype are suggestive of defective actin polymerization during the “dumping” of nurse cell contents into the late oocyte: ring canals have collapsed, actin filaments along the nurse cell plasma membrane are discontinuous (Figure 3, F′ and F′′), and the radial arrays of actin cables emanating from the nurse cell plasma membrane are less abundant than the wild type (Cooley *et al.* 1992; Murphy and Montell 1996; Li *et al.* 1999).

One complementation group (*11R5*, *13P2*, *13P10*, and *13P13*) has lesions in the *rasp* gene (Table 2), which encodes a Protein-cysteine *N*-palmitoyltransferase (Chamoun *et al.* 2001). All alleles generate eggs with fused appendages, indicative of reduced Grk signaling, although levels and localization of *grk* mRNA appear normal. Rasp is required for the palmitoylation and activation of Spitz, an epidermal growth factor receptor ligand, and has been proposed to be also required for Grk activity (Miura *et al.* 2006). Grk protein is diffusely localized in the cytoplasm of mutant oocytes, rather than adjacent to the cortex as in the wild type (Figure 3, G and H). This result suggests that lack of posttranslational modification causes Grk to be mistrafficked and not fully activated.

### *elg1* is required for *grk* transcript localization and nurse cell endoreplication

**Figure 4:**
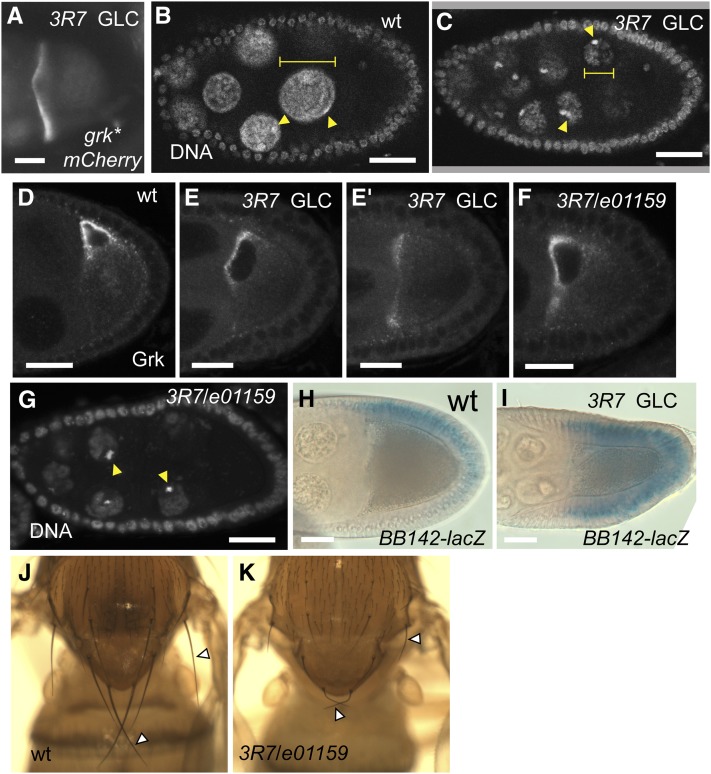
*elg1* is required for *grk* mRNA localization and nurse cell DNA endoreplication. (A) grk*mCherry localization, spreading along the anterior periphery in *elg1^3R7^* stage 8−9 oocytes. (B, C, and G) DAPI staining of stage 8 egg-chambers showing the *elg1* nurse cell nuclei [(B, G) are smaller than wild-type (diameters; yellow bars]. Mutant nurse cell nuclei include an increased proportion of condensed heterochromatin (arrowheads), which is usually relatively underreplicated during endoreplication (Dej and Spradling 1999). (C) *elg1^3R7^* germline clone (GLC) and (G) *3R7/e01159* egg-chamber. (D−F) Anti-Grk staining of the wild-type (D) and *elg1* mutant stage 8−9 egg-chambers, showing that Grk protein localizes across the entire anterior end in *elg1^3R7^* germline clone oocytes (E and E′; two different planes of germline clone) and *3R7/e01159* egg-chamber (F). (H, I) X-Gal staining of *BB142-lacZ* enhancer trap in wild-type (H) and *3R7* germline clone egg-chambers (I), showing ectopic (ventral; bottom) staining in the latter. (J, K) Macrochaetae (arrowhead) are shorter in *3R7/e01159* scutellum (J) than in the wild type (I). Scale bars = 20 μm.

*grk* transcripts mislocalize along the entire anterior margin of *3R7* oocytes (Figure 4A). *3R7* also affects nurse cell DNA endoreplication: mutant nurse cell nuclei are smaller and apparently underreplicated (Figure 4, B and C). Translation of the mislocalized transcripts is not repressed, and so Grk protein accumulates anteriorly (Figure 4, D, E, and E′), where it drives ectopic expression in follicle cells of a Grk-responsive *lacZ* enhancer-trap (*BB142*; Figure 4, H and I; Schüpbach and Roth 1994). In these and other respects, the *3R7* oocyte phenotype differs from that associated with a DNA-damage response (see following section).

*3R7*, which is represented by a single allele, maps by SNP-recombination between 70E and 73E, within which WGS identified a W212 > stop nonsense mutation that would truncate most of the 1162 amino acid protein in *CG16838*, a *Drosophila* homolog of yeast *elg1* (Table 2). The mutation is likely to eliminate Elg1 activity since *3R7* is not complemented for female fertility, Grk protein localization, or nurse cell DNA replication by *e01159*, a *piggybac* insertion into the coding region of *CG16838* (Figure 4, F and G), confirming that the causative mutation indeed lies in *elg1*.

Elg1 is a factor that binds to and loads proliferating cell nuclear antigen onto replicating DNA (reviewed in Aroya and Kupiec 2005) and is required for maintaining genome stability in yeast and human cells (Kanellis *et al.* 2003; Sikdar *et al.* 2009). Homozygous *3R7* flies as well as *3R7*/*e01159* mutant flies are viable (albeit female sterile) and develop without significant difference in adult body size. Also, their nota display shortened macrochaetae (Figure 4, J and K), whose lengths also depend on endoreplication (Weng *et al.* 2003). This phenotype suggests that *elg1* is also required for the growth of other cell types that undergo endoreplication.

### *armi* is required for somatic ovarian development

**Figure 5:**
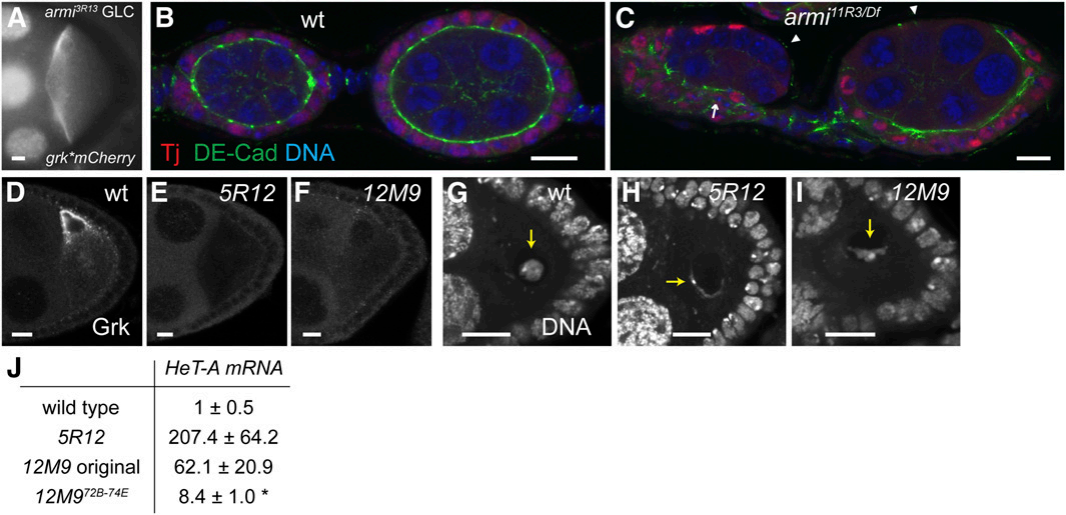
Mutants affecting transposon silencing. (A) grk*mCherry localization, spreading along the anterior periphery in *armi^3R13^* stage 8−9 oocytes. (B) wild-type and (C) *armi^11R3^ / Df(3L)E1* ovarioles. Costainings of Traffic jam (Tj, marker for follicle cells), DE-Cadherin (DE-Cad), and DNA (DAPI). Follicle cells in *armi* mutant are disorganized (arrow) and fail to encapsulate the germline cyst (arrowheads). (D−F) lack of Grk protein expression in mutant stage 8−9 egg-chambers. (G-I) DAPI staining in stage 3-5 egg-chambers showing distorted oocyte karyosomes (arrows). (D and G) wild type. (E and H) *5R12* germline clone. (F and I) *12M9* germline clone. (J) Mean levels of *HeT-A* transcripts (± SD) are elevated in mutant egg-chambers compared with the wild type. *P < 0.001. Scale bars = 10 μm.

Other newly induced mutations appear to affect transposon silencing. Complementation analysis and DNA sequencing showed that 8 of the 11 mutants affecting *grk* mRNA localization are allelic to *armitage* (*armi*), which encodes a putative RNA helicase required for the transposon silencing by Piwi-interacting RNAs (piRNAs), small RNAs that associate with the Piwi protein (Cook *et al.* 2004; Malone *et al.* 2009). Like other piRNA pathway mutations, *armi* oocytes show phenotypes associated with the response to DNA damage caused by excessive transposon expression, including failure to restrict anterior *grk* mRNA localization to a dorsal corner (Figure 5A) due to a misorganized MT cytoskeleton, silencing of Grk translation, and disruption of the karyosome (not shown; Klattenhoff *et al.* 2007; Pane *et al.* 2007).

Armi has been shown to function together with Piwi and Fs(1)Yb (Saito *et al.* 2010; Qi *et al.* 2011). However, previous *armi* alleles (*armi^72.1^*; *armi^1^*) did not show the abnormal follicle cell development and defective germline stem cell reproduction seen in *piwi* mutant females (Cox *et al.* 1998; Cook *et al.* 2004). Our results show that this is probably due to residual somatic *armi* activity. Females hemizygous for our novel, strong *armi* alleles produce no mature eggs (Table 3). Ovaries of those females display a variety of defects in follicle cell organization, including a failure to encapsulate the germline and formation of a multilayered epithelium (Figure 5, B and C). Similar phenotypes are seen in another piRNA pathway mutant, *vreteno* (Zamparini *et al.* 2011).

### CG33522 (Scaf6/Cherp) is required for transposon silencing

The nonallelic *grk* mRNA mislocalization mutants *5R12* and *12M9* both show *grk* mistranslation and a defective karyosome indicative of a DNA damage response (Figure 5, E, F, H, and I). To confirm that the novel mutations affect transposon silencing, we measured transcription of telomeric transposon *HeT-A*, whose expression is sensitive to piRNA-mediated silencing (Pane *et al.* 2007; Li *et al.* 2009). For both mutations, *HeT-A* mRNA levels in the ovaries are greatly increased (Figure 5J), indicating that piRNA silencing is indeed disrupted.

SNP-recombination analysis showed that *12M9* is due to at least two interacting lesions. In summary, a distal mutation between 72B−74E causes weaker transposon activation (Figure 5J), which is enhanced by a proximal mutation between 74E−80B that also reduces germ cell maintenance such that no developing egg-chambers remain 10 d after clone induction. The enhancer mutation had no other apparent maternal phenotype in the absence of the distal mutation and so was not investigated further.

Sequencing *12M9* revealed the induction of a nonsense mutation (Q560 > stop) in *CG33522* (at 73E5), the *Drosophila* homolog of human *SR-related CTD associated factor 6* (*scaf6*)/*calcium homeostasis endoplasmic reticulum protein 6* (*cherp*) (Will *et al.* 2002; Jurica and Moore 2003). Animals transheterozygous for the *12M9* chromosome and *Df(3L)ED4674*, a deficiency lacking 73B5−73E5, live until the late pupal stage but rarely eclose. Viability and fertility are rescued by a 20-kb transgene covering *scaf6* (Pacman genomic clone *CH322-104B15*; Venken *et al.* 2009), implying that the *scaf6* mutation is indeed causative.

### *saturn* is due to combined inactivation of *klc* and the piRNA pathway

**Figure 6:**
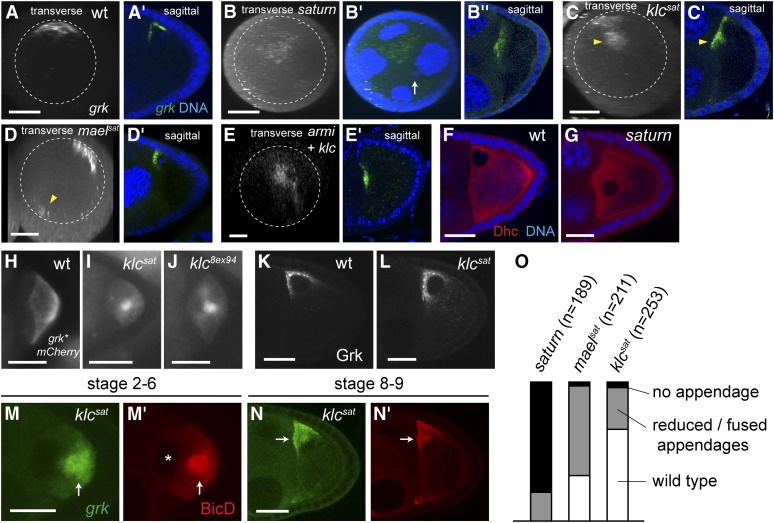
Anterior-central *grk* mRNA localization in *saturn* is due to joint inactivation of *klc* and piRNA pathway. (A−E) Sagittal views as well as transverse projections of a series of z-stack confocal images of endogenous *grk* mRNA visualized by *in situ* hybridization in wildtype (A), *saturn* (B), *klc^sat^* (C), *mael^sat^* (D), and *armi^3R13^ klc^8ex94^* double mutant (E) stage 8−10 egg-chambers. Nuclear DNA in the *saturn* oocyte is marked by arrow (B′), showing that the majority of *grk* mRNA is not associated to the nucleus. Approximately one-half the *grk* mRNA (arrowhead, C) is internalized in *klc^sat^* germline clones. *grk* mRNA is also seen in the opposite side of the anterior periphery (arrowhead, D) in *mael^sat^* germline clones. (F, G) Anti-Dhc staining in wild-type (F) and in *saturn* (G) stage 8−9 egg chambers. Dhc is excluded from the nucleus, thus showing the mispositioned nucleus. (H, I, J) grk*mCherry localization in stage 6 oocytes. (H) wild-type (wt), (I) *klc^sat^*, and (J) *klc^8ex94^* showing internally localized *grk* mRNA in *klc* mutants. (K, L) Anti-Grk staining in stage 8−9 egg-chambers. (K) wildtype (wt). (L) *klc^sat^*, showing that Grk translation is not disrupted in *klc* mutant. (M, N) Localization (arrows) of grk*mCherry (*grk*, M and N) and the dynein cofactor, BicD (M′ and N′) indicates that dynein-dependent *grk* mRNA transport is not affected in *klc^sat^* germline clone. BicD and *grk* mRNA are excluded from the oocyte nucleus, showing a posteriorly mislocalized nucleus before its migration to the dorsoanterior corner. (O) Frequencies of abnormal or absent dorsal appendages in mature eggs of indicated genotypes. Scale bars = 5 μm in (M) and 20 μm in the rest.

The final mutation (*5R12*, which we named “*saturn*”), also affects transposon expression but differs phenotypically from other piRNA pathway mutants in that *grk* mRNA and the nucleus localize anteriorly but not in a corner (Figure 6, A−G). SNP-recombination mapping combined with WGS showed that *saturn* is a synthetic mutation: *klc* (AG > GG in the splice-acceptor of the second intron, which probably inactivates the gene due to skipping of the 121 base exon 3 and a translational frame shift), combined with *maelstrom* (*mael*; E131 > R), which encodes a piRNA pathway component that is required for *grk* mRNA localization and MT polarization (Clegg *et al.* 1997; Findley *et al.* 2003). We call these individual alleles *klc^sat^* and *mael^sat^*, respectively (see Figure S2 for mapping details).

To check whether combined reduction of piRNA and *klc* activities explains the *saturn* phenotype, we combined a different null *klc* allele with a mutation in a different piRNA pathway gene. *klc^8ex94^ armi^3R13^* double-mutant oocytes show a *saturn*-like phenotype with a complete lack of cortical localization of *grk* mRNA in stage 10 oocytes (Figure 6E), indicating that the *saturn* phenotype is indeed caused by the *klc^sat^ mael^sat^* mutations.

### MT organization is altered in *klc* mutant oocytes

*grk* mRNA is normally tightly associated with the oocyte cortex but, in stage 9 *klc^sat^* oocytes, approximately one-half of the *grk* mRNA is somewhat internally localized (Figure 6C), a phenotype that is evident from as early as stage 2 (Figure 6I). A similar phenotype is seen in *klc^8ex94^* germline clones (Figure 6J; Gindhart *et al.* 1998).

Levels of Grk protein appear unaffected in *klc^sat^* oocytes (*cf*. Figure 6, K and L), indicating that a DNA damage checkpoint has not been triggered. Nevertheless, the mutant eggs are somewhat ventralized, showing that Grk signaling activity is reduced (Figure 6O). Presumably, *grk* mRNA mislocalization causes Grk to be less efficiently processed or secreted.

Internally mislocalized *grk* mRNA in *klc* mutant oocytes could be due either to impaired RNA transport or to MT misorganization. Dynein cofactors Egl and BicD have been previously shown to be in the *grk* mRNA transport complex (Delanoue *et al.* 2007), and most *grk* mRNA colocalizes with BicD from early stages until at least stage 9 (File S4). BicD and *grk* transcripts still colocalize in *klc^sat^* mutant oocytes (Figure 6, M, M′, N, and N′), indicating that dynein-mediated transport of *grk* mRNA is still active in *klc* mutant oocytes and the *grk* mRNA mislocalization is due to an altered MT cytoskeleton.

### *klc* and piRNA pathways are required for the clustering of oocyte centrosomes

**Figure 7:**
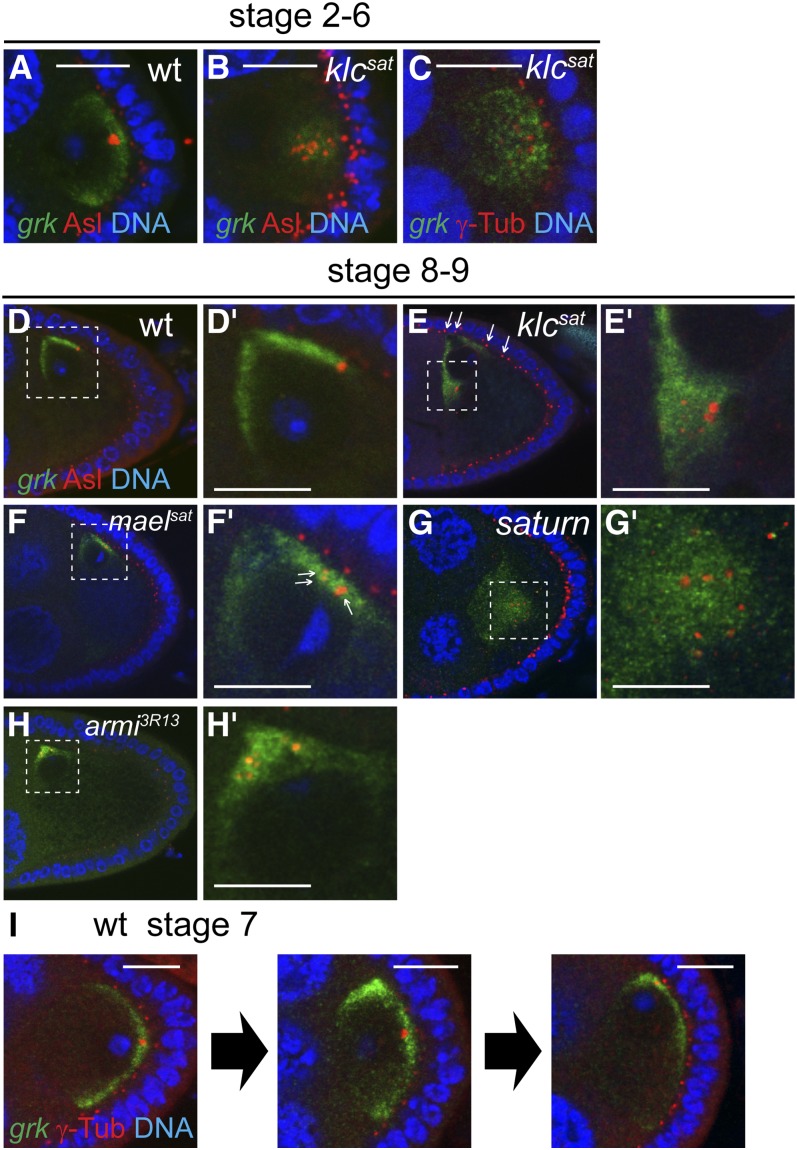
Disrupted centrosome clustering in *klc* and piRNA pathway mutant oocytes reveals two modes of microtubule organizations involved in *grk* mRNA localization. Costaining of grk*mCherry (*grk*) and centrosome components Asterless (Asl, A, B, D-H) or γ-Tubulin (γ-Tub, C, I) in wild-type (A, D, I), and *klc^sat^* (B, C, E), *mael^sat^* (F), *saturn* (G), and *armi^3R13^* (H) germline clones. D′-H′ are the enlarged images of selected square in D−H, respectively. Centrosome components are dispersed both in *klc* (B, C, and E) and piRNA pathway mutants (*mael* in F′ and *armi* in H′) and are internally misplaced in *klc* mutant, like *grk* mRNA (E). Arrows indicate faint centrosomes deriving from follicle cells (E). Centrosome components are more dispersed in *saturn* (G′) oocyte than in *klc* (E’) and *mael* (F′) mutant oocytes alone. (I) Snapshots of stage 7 wild-type egg-chambers, showing that dorsal-anterior localization of *grk* mRNA precedes the centrosome migration. Scale bars = 10 μm.

During early oogenesis, nurse cell centrosomes are exported to the prospective oocyte where they form a single, large cluster (Bolivar *et al.* 2001). Our results show that efficient formation of this aggregate is dependent on kinesin activity. Staining *klc* mutant oocytes for the pericentriolar proteins Asterless and γ-tubulin reveals 10−20 foci clustered posteriorly at stage 2 and anteriorly but somewhat internally from stage 8 (Figure 7, A−E and E′). Costaining for Asl. and grk*mCherry shows that internally localized *grk* mRNA in *klc* mutant oocytes follows the dispersed centrosomes (Figure 7, B, C, and E), either as a consequence of displacing the centrosomes, or because related cues drive *grk* RNA and centrosomal localization.

Centrosomes are also less tightly clustered in *mael^sat^* oocytes (Figure 7, F and F′), and in *armi^3R13^* mutant oocytes (Figure 7, H and H′), suggesting that defects in the piRNA pathway also lead to this phenotype. Centrosomes are even more dispersed, and *grk* mRNA is even less focused in *saturn* mutant oocytes (Figure 7G′), illustrating that loss of function of *klc* and defective piRNA pathway have additive effects.

### Breakage of dorsoventral asymmetry is associated with centrosome-independent transport

Although most *grk* transcripts localize around the centrosomes, a significant proportion accumulates in a centrosome-free domain adjacent to the nucleus. In stage 8−9 wild-type oocytes, the centrosome lies dorsal to the nucleus, yet some *grk* RNA lies anterior to the nucleus (Figure 7D). In *klc* oocytes, a population of *grk* mRNA still localizes to the dorsal cortex, away from the centrosomes, which now lie ventral to the nucleus (Figure 7E). Together, these results suggest that a population of *grk* mRNA is transported on MTs that nucleate from the cortex.

To test the relationship between *grk* mRNA localization and centrosomes, we focused on stage 7, when the site of *grk* localization changes from posterior to dorsoanterior (Neuman-Silberberg and Schüpbach 1993). Many wild-type stage 7 oocytes show an intermediate pattern of *grk* RNA localization in which transcripts lie both dorsoanteriorly and posteriorly (Figure 7I, middle image). We find that the centrosome is still at the posterior in 60% (19/31) of such oocytes, indicating that *grk* relocalization usually precedes that of the centrosome. Together, these results argue that breakage of radial symmetry is not driven by centrosome migration.

Early, centrosome-independent *grk* localization reflects asymmetric RNA transport rather than differential anchoring of RNA because BicD, which is involved in transport but not anchoring of *grk* mRNA (Delanoue *et al.* 2007), also accumulates dorsoanteriorly (Figure S3B). These results suggest that breakage of radial symmetry and formation of a dorsoventral axis arises from centrosome-independent reorganization of the MT cytoskeleton (see *Discussion*).

## Discussion

### Rapid gene identification by SNP mapping combined with WGS

WGS has revolutionized methods for identifying mutational lesions and has been applied to many organisms, including humans (Sarin *et al.* 2008; Blumenstiel *et al.* 2009; Irvine *et al.* 2009; Berger *et al.* 2012). Such sequencing is readily affordable for *Drosophila*. A single multiplexed pool of 5−10 mutants yields ~3.3 Gb of mutant sequence data with which we could reassemble >99% of the euchromatic region of chromosome 3L, reducing the cost to about $150 per mutant (unpublished data R. Hayashi and S. Horswell).

By using two-stage recombinational mapping with SNP markers in a single generation, we were also able to map causative molecular lesions very rapidly, thereby rendering unbiased EMS-based genetic screens practical, even for complex phenotypes (Figure 2 and File S3). The first stage uses telomeric and centromeric SNPs to identify recombinants, and to generate linkage data that map the causative locus approximately. A second stage of “digital” mapping with internal SNPs, performed without additional crosses, locates the lesion to a minimal region. A related recombinational strategy using dominant markers and WGS has been proposed by Sapiro *et al.* (2013). Our strategy would seem to be at least as rapid, requires fewer recombinants, and maps the mutations more accurately. It is also more flexible as it is not prescriptive as to the tester chromosome, which needs only to be unrelated to the mutagenized one (particularly important for phenotypes that are sensitive to genetic background).

SNP mapping also makes it worthwhile following up synthetic phenotypes, as demonstrated by our mapping and analysis of the *saturn* double-mutant (Figure S2). Such occurrences are surprisingly frequent due to the efficiency of EMS mutagenesis; the phenotypes of two of the four single-allele mutants in our 3L screen result from two interacting mutations, as do approximately one-third of single-allele mutants in the equivalent 3R screen (unpublished data).

### Kinesin-1 is required for centrosome clustering and cortical localization

By analyzing *klc^sat^* null oocytes, we have shown that *klc* activity is required for cortical *grk* mRNA localization and, later, also for efficient dorsoanterior localization of the RNA (Figure 6, C, I, L, and O). BicD is similarly affected in the mutant oocytes, demonstrating that the *grk* mislocalization is due to alterations of MT cytoarchitecture (Figure 6, M and N). Kinesin-1 is also needed for the clustering that follows the migration of nurse cell centrosomes into the pro-oocyte. Centrosome aggregation is never seen in *klc* mutant oocytes (Figure 7, B, C, and E), suggesting that *klc* is required to initiate a cluster rather than only for its stabilization.

The latter phenotype is probably due to reduced dynein activity, which can mediate the sliding of antiparallel MTs that is required for centromere clustering (Goshima *et al.* 2005; Braun *et al.* 2009; Fink *et al.* 2009; Gatlin and Bloom 2010). Evidence for the linkage of kinesin and dynein activities comes from studies showing that mid-stage *Khc* mutant oocytes have reduced Dhc levels (Loiseau *et al.* 2010) and that *klc* embryos are sensitive to reduced dynein activity (Duncan and Warrior 2002). Dynein activity is also altered in piRNA pathway mutants (Navarro *et al.* 2009), in which centromeric clustering is also impaired (Figure 7, F and H).

Clustering may also depend on mutual tension between centrosomes, the cortex and the nucleus, which is likely to be weakened in *klc* mutants, as evidenced by the relative displacement of the dispersed centrosomes away from the cortex (Figure 7, B, C, and E). Such tension, generated from MT attachment to the cortex and the kinetochore, is thought to be responsible for the clustering of supernumerary centrosomes in cancer cells (Kwon *et al.* 2008; Leber *et al.* 2010).

### Centrosome-independent transport of *grk* mRNA suggests breakage of axial symmetry via MT self-organization

The dorsoanterior localization of *grk* mRNA and the oocyte nucleus marks the formation of a single organizing center and represents a key breakage of radial symmetry. This center is clearly a major site of MT minus-end nucleation, as marked by BicD staining (Figure S3).

The association between centrosomes and the major domain of *grk* localization in both wild-type and *klc* oocytes (Figures 7, A−E) indicates that centrosomes promote nucleation of many of the MTs used to transport *grk* mRNA. However, a significant proportion of *grk* transcripts localizes in a distinct domain that is not associated with the centrosomes (anteriorly in wild-type oocytes; dorsally in *klc* oocytes; Figure 7, D and E), implying that centrosome-independent MTs are also involved in *grk* mRNA localization. Such acentrosomal MTs could be nucleated by the oocyte cortex and the nucleus. Injection of *bicoid* transcripts into the oocyte and MT regrowth experiments have suggested that MTs can grow from the anterior and lateral oocyte cortex and from subnuclear regions (Cha *et al.* 2001; Januschke *et al.* 2006; Parton *et al.* 2011) and the dynein component Dynamitin localizes around the oocyte nucleus (Januschke *et al.* 2002).

These two domains of *grk* transcript localization largely overlap throughout oogenesis in wild-type oocytes, except during stage 7 when MT reorganization and nuclear migration take place (Figure 7I). The trigger for this repolarization is an unknown signal from the posterior follicle cells (Gonzalez-Reyes and St. Johnston 1998), but it is clearly accompanied by extensive MT remodeling. Zhao *et al.* (2012) have suggested that the posterior-lying oocyte nucleus is “pushed” anteriorly by centrosomally nucleated MTs. This mechanism accounts for anterior movement of the nucleus but does not explain why it ends up in a corner rather than at the center of the anterior surface or the formation of a single dorsoventral axis. The oocyte centrosome cannot be the basis of such uniqueness because dorsoanterior *grk* mRNA localization usually precedes anterior centrosome migration (Figure 7I), suggesting that the behavior of noncentrosomal MTs are key to defining the unique axis.

How might noncentrosomal MTs generate a unique dorsoventral axis? Jaramillo *et al.* (2008) have described a transient phase of dorsoventral patterning in which *grk* mRNA is partly localized in an anterior ring. To resolve such radial symmetry requires selective stabilization of a single dorsoventral organizer site at the expense of others which, in turn, implies a form of positive feedback that favors the final site.

Such feedback might be driven by MT self-organization, which has been observed in several other contexts (Wasteneys and Ambrose 2009; Mogilner and Craig 2010; Sumino *et al.* 2012). The oocyte itself is not radially symmetric (due to distortions imposed on its anterior surface by the nurse cells; File S5), and this spatial constraint may restrict the number of dorsoventral MT foci, and might explain why the reduced nurse cell size of *elg1* egg-chambers affects *grk* mRNA localization (Figure 4). Further genetic approaches, as well as direct *in vivo* visualization of the MT and motor dynamics, should lead to a better understanding of how MT reorganization and anterior nuclear migration are achieved during oogenesis.

## Supplementary Material

Supporting Information
