# Supplementary material for: A Genetic Screen Based on in Vivo RNA Imaging Reveals Centrosome-Independent Mechanisms for Localizing gurken Transcripts in Drosophila
Source: G3 (Bethesda). 2014 Feb 14;4(4):749–60. doi: 10.1534/g3.114.010462 (PMC4059244; doi:10.1534/g3.114.010462)
Supplement: Supporting Information [file supp_4_4_749__index.html]

A Genetic Screen Based on in Vivo RNA Imaging Reveals Centrosome-Independent Mechanisms for Localizing gurken Transcripts in Drosophila — Supporting Information 

# A Genetic Screen Based on *in Vivo* RNA Imaging Reveals Centrosome-Independent Mechanisms for Localizing *gurken* Transcripts in *Drosophila*

## Supporting Information for Hayashi *et al.*, 2014

**Files in this Data Supplement:**

- Supporting Information - Files S1-S5 (PDF, 254 KB)
- File S1 - Methods (PDF, 183 KB)
- File S2 - An example of KASPAr results. (PDF, 137 KB)
- File S3 - *Saturn* is caused by two mutations that are mapped to 69C-70C (*saturndist*) and 72B-80B (*saturnprox*). (PDF, 162 KB)
- File S4 - *grk* mRNA colocalizes with BicD throughout oogenesis. (PDF, 173 KB)
- File S5 - Anterior of stage 8 oocyte lack axial symmetry. (.mov, 2 MB)
