# Supplementary material for: A Genetic Screen Based on in Vivo RNA Imaging Reveals Centrosome-Independent Mechanisms for Localizing gurken Transcripts in Drosophila
Source: G3 (Bethesda). 2014 Feb 14;4(4):749–60. doi: 10.1534/g3.114.010462 (PMC4059244; doi:10.1534/g3.114.010462)
Supplement: Supporting Information [file supp_g3.114.010462_010462SI.pdf]

G3: Genes | Genomes | Genetics

A genetic screen based on in vivo RNA imaging reveals centrosome-independent mechanisms for localizing *gurken* transcripts in *Drosophila*

Rippei Hayashi*, S Mark Wainwright*, Sophie J Liddell*, Sheena M Pinchin*, Stuart Horswell† and David Ish-Horowicz*,[1]

*Developmental Genetics Laboratory and †Bioinformatics Unit, London Research Institute, 44 Lincoln's Inn Fields, London WC2A 3LY, UK

[1]Corresponding author: David Ish-Horowicz, MRC Laboratory for Molecular Cell Biology, University College London, Gower St, London WC1E 6BT, UK. E: d.horowicz@ucl.ac.uk

DOI: 10.1534/g3.114.010462

File S1

METHODS

Fly stocks

The following Drosophila melanogaster fly stocks were used: *w; P{FRT(w[hs])}2A*; *w; P{ovo^{D1-18}}3L1 P{FRT(w[hs])}2A; y w P{hsFLP}1; w/Dp(2;Y)G, P{hs-hid}Y; ru h th st cu sr e ca (rucuca), w; Dr^{Mio}/TM3, P{gal4-twi.G} P{UAS::2xEGFP}AH2.3, Sb Ser* from the Bloomington stock center; two copies of *grk(MS2)$_{12}$* transgenes on 2nd chromosome from Trudi Schüpbach; *nos::MCP-mCherry* (this study). (In the course of these experiments we mapped the insertion site of *P{hsFLP}1* to just upstream of the *merlin* gene at 18E.) As recombinants had to include an *FRT* site for phenotypic analysis, we generated a recombinant *ru h th st FRT2A* chromosome from the sequenced *FRT2A* and *rucuca* chromosomes (Figure 2C), using SNP markers to identify a line which had recombined closest to the *FRT* site.

Other fly stocks used: *Df(3L)E1* for *armi*; *Df(3L)ED4674* for *scaf6*; *Df(3L)Exel6087* for *half pint*; *qm$^{L14.4}$*; *klc^{8ex94}* from Bloomington stock centre; *PBac{RB}CG16838^{e01159}* from Harvard stock collection; *spn-C^{094}* from Daniel St. Johnston; *Tsc2$^{192.1}$* from Nic Tapon; *BB142-lacZ* enhancer trap from Trudi Schüpbach.

Pacman genomic clone *CH322-104B15* was obtained from BACPAC Resource Center in Oakland and the transgenic flies were made by BestGene Inc. using the *attP* site at 28E7 (*PBac{y+-attP-3B}VK00002*; Venken *et al.* 2009).

Enquiries regarding mutant stockes and the sequence analysis software described in this paper should be directed to Rippei Hayashi <rippei.hayashi@imba.oeaw.ac.at>.

Construction of transgene

The promoter and 5'UTR of *nanos* gene (*nanos-5'*) were amplified from *nos-Gal4-VP16* plasmid (Van Doren *et al.* 1998) and subcloned into *pBluescript-II* using *Bam*HI and *Kpn*I sites. A linker sequence was added downstream of *Kpn*I to make *Not*I site. *BamHI-nanos-5'-KpnI-NotI* was cut and cloned into *pCaSpeR-2* using *Bam*HI and *Not*I sites. *K10-3'*, which contains the region that stabilises the transcript in germline, but lacks the stem-loop that localises the transcript into the oocyte, was amplified from *pUASp* plasmid (Serano *et al.* 1994; Rorth 1998) and inserted downstream of *nanos-5'* using *Not*I and *Eco*RI sites to make *pCaSpeR-2-nanos-5'-KpnI-NotI-K10-3'*. *NLS-MCP* was amplified from *hsp83-MCP-GFP* flies (Forrest and Gavis 2003) and subcloned into *pUASp* using *Kpn*I and *Bam*HI sites. *mCherry* (Shaner *et al.* 2004) was inserted downstream of *NLS-MCP* using *Bam*HI and *Not*I sites. The sequence encoding FLAG peptide was added to the C-terminal of mCherry by PCR and *KpnI-NLS-MCP-mCherry-FLAG-blunt* was ligated to *pCaSpeR*-2-*nanos-5'-KpnI-NotI-K10-3'*, which had been cut by *Not*I and blunted with the Klenow fragment of DNA polymerase, followed by *Kpn*I digestion, to finally obtain *pCaSpeR*-2-*nanos-5'-MCP-mCherry-K10-3'*. *nanos-5'-MCP-mCherry-K10-3'* transgenic flies were generated by standard protocol by selecting w^+.

Mutagenesis and screen method

1-3 days old isogenic *FRT2A* males were starved for 2-4 h before being fed overnight with 25 mM EMS (Sigma) in 1% sucrose. Mutagenized males were mated for three days with *w; TM3, Sb/TM6B, Tb* virgin females after 24 h recovery from mutagenesis. Single *w; *FRT2A/TM3 Sb or TM6 Tb* virgin females were mated with *hsFLP, nos::MCP-mCherry; grk(MS2)$_{12}$; P{ovo^{D1}} FRT2A/Sb or TM3, Ser* tester males (* indicates mutagenized chromosome.). Tester males were maintained by crossing them to *hsFLP, nos::MCP-mCherry; grk(MS2)$_{12}$; Sb/TM3, Ser* virgin females. Progeny were heat-shocked for 3 h in a 37°C incubator on each of three consecutive days during larval and pupal stages.

Preparation of genomic DNA for WGS

Genomic DNA was prepared from adult flies homozygous for the isogenised reference chromosomes (*FRT2A* and *rucuca*), and viable mutated *FRT2A* chromosomes. For recessive lethal mutated *FRT2A* chromosomes, homozygous embryos or larvae were selected using *TM3, Sb Ser, twi::Gal4, UAS-EGFP* (Bloomington No. 6663).

DNA was prepared using a modification of standard protocols. Briefly, 50-100 frozen adult flies were ground in 300 μl of buffer A [10 mM Tris-HCl, pH 7.5, 60 mM NaCl, 10 mM EDTA, 0.15 mM spermine, 0.15 mM spermidine, 5% sucrose, and 0.5% Triton X-100]. Cuticular debris and floating lipid were removed by centrifugation, and nuclei purified by pelleting and washes in buffer A. They were resuspended in 300 μl of buffer A, and lysed with 300 μl of 2% w/v N-lauryl sarcosine, 0.1 M EDTA, 5% sucrose, and incubation at 65°C for 45 min. Nucleic acids were extracted with phenol/chloroform and chloroform, and precipitated with sodium acetate/2-propanol. About 50 μg of DNA was obtained from 100 flies.

To purify embryonic or larval DNA, several hundred 24-36 h old unhatched eggs or 1st/2nd instar larvae were homogenized in buffer B [10 mM Tris-HCl, pH 8.0, 25 mM NaCl, and 1 mM EDTA. 1% SDS and 0.1 mg/ml of proteinase K (Roche) were added before use.] and incubated at 37°C for 1 h. SDS was precipitated with 1 M

NaOAc, and nucleic acid was recovered from the supernatant with phenol/chloroform and 2-propanol precipitation. The dried pellet was dissolved in TE containing 0.1 mg/mg RNase A (Qiagen) and incubated at 37°C for 1 h. RNase A was removed with phenol/chloroform, and DNA was precipitated with NH_4OAc and 2-propanol. About 1 μg of DNA was obtained from about 300 embryos or 100 larvae.

Illumina WGS and data analysis

1 μg of purified DNA was sheared to ~300 bp in 120 μl of TE using a Covaris S2 sonicator (Covaris, Wobum, MA, USA), and libraries for Illumina sequencing were prepared according to the manufacturer's instructions. The size and the quality of DNA in the library were checked by BioAnalyser 2100 (Agilent, Santa Clara, CA, USA). Flowcell cluster formation on a cluster station and 72 bp paired-end or 36 bp single-end sequencing by synthesis on the Genetic Analyser IIx were performed according to the manufacturer's protocol. Seven and one lanes of 72 bp paired-end sequencing were run for *FRT2A* and the mutagenised lines, respectively. This sequencing showed that FRT2A is inserted at around 22,865k (only about 150kb proximal to *mael*). Two lanes of 36 bp single-end sequencing were run for *rucuca*.

Raw reads in FASTQ format, which were generated by the Illumina sequencing pipeline without purity filtering, were aligned to the DM3 build (ftp://hgdownload.cse.ucsc.edu/goldenPath/currentGenomes/Drosophila_melanogaster/bigZips/) of the *D. melanogaster* genome using the bwa 0.5.9 program (Li and Durbin 2009) with a maximum mismatch threshold of 2 (and a seed length of 72 or 36) and all other parameters left at the defaults. From a typical single lane of 72 bp paired-end sequencing, we obtained a total sequence of 3.3±0.5 GB (average ± S.D. of 12 sequencing runs, ~20 genome equivalents). The output Sam files were converted to pileup format using Samtools 0.1.16 (Li *et al.* 2009).

Candidate variants were identified using in-house code (details available on request), which extracts any site that carries an allele present in either the parental *FRT2A* or mutagenised line, but not both, subject to the following criteria: minimum of 7 reads in both the parental *FRT2A* and mutagenised lines, minimum of 5 instances of the "variant" allele. For frequent reads, the variant allele must also constitute at least 35% of the total reads at that site (to reveal poorly-called variants). Lowering of these threshold criteria did not increase genome coverage but increased the frequency of variant calls, suggesting that these were mainly sequencing errors. Variant positions were annotated as "transcribed region", "coding sequence", "exonic" or "intron" according to the UCSC-RefSeq database <http:// genome.ucsc.edu/>. Sequence data are accessible via Sequence Read Archive (SRA) accession number SRP037983.

SNP mapping

The length of the euchromatic region of each of the five major Drosophila chromosome arms is between 21.1 and 27.9 Mb (release 5, <http://flybase.org/>). The standard EMS protocol induces one mutation per ~400 kb, corresponding to 50-70 EMS-induced mutations per chromosome arm, and to 9-13 mutations in protein-coding regions (Misra *et al.* 2002).

To identify causative mutations, 48-96 **?FRT2A/TM3, Sb or TM6, Tb* F2 virgin recombinant females were individually mated with tester males carrying *TM3, Ser* for phenotypic retesting, SNP mapping, and to make stocks of informative recombinants (Figure 2C). DNA was extracted from individual **?FRT2A/TM3, Ser* F3 males by grinding in 50 μl Buffer B, and incubated for 1 h at 37°C followed by 5 min at 95°C. 0.5 μl was used for KASPAr allelic-discrimination PCR (KBiosciences; <http://www.kbioscience.co.uk/>), which uses PCR primers tagged with homologies to beacon DNA probes that fluoresce (FAM or VIC fluorochromes) only when extended. DNA amplification and genotyping were performed in microtiter plates (see Supplementary File S2 for a typical result). Informative F3 females (showing recombination between proximal and distal SNPs) were phenotyped as in the primary.

SNPs for mapping *11R2* and *saturn*:

61A: C97,297 in reference genome and *rucuca*; "G" in *FRT2A*.
66F: A8,995,643 in reference genome and *rucuca*; "T" in *FRT2A*.
69C: A12,553,021 in reference genome and *rucuca*; "T" in *FRT2A*.
70C: G13,598,030 in reference genome and *FRT2A*; "C" in *rucuca*.
72B: A15,920,131 in reference genome and *FRT2A*; "C" in *rucuca*.
75F: T18,997,112 in reference genome and *rucuca*; "C" in *FRT2A*.

RNA extraction and qRT-PCR:

Germline clones of parental *FRT2A* and mutant *FRT2A* chromosomes were generated as described, and females were dissected 8-10 days after the heat-shock. RNA was extracted from 10-15 pairs of ovaries in each preparation using RNeasy columns (Qiagen). qRT-PCR was performed using Superscript III Platinum for SYBR-green one-step qRT-PCR (Invitrogen). Primers used are (5'-3'): *HetA*, GACCCCCTCTCACTCACCAAT and GAGGTGGACGGAGGAGAAGAC; CGCGATCCTTGAAAATCTTTG and CGAACTCCTGCATCACATCCT for *nanos*.

Antibody staining, in situ hybridization and X-gal staining:

Antibody staining was performed using standard techniques. Ovaries were fixed for 10 min in 1% formaldehyde for Asterless or γ-Tubulin detection, and 4% formaldehyde was used for other antibodies. Antibodies used were mouse IgG1 anti-Orb (1:200, DSHB 4H8), mouse IgG2a anti-β-Galactosidase (1:1000, Promega), mouse anti-Gurken (1:200, DSHB 1D12), guinea pig anti-Traffic Jam (1:5000, gift from Dorothea Godt), rabbit anti-Asterless (1:500, gift from Jordan Raff), mouse anti-BicD (1:200, DSHB 4C2), mouse anti-Dynein heavy chain (1:200, DSHB 2C11-2), mouse anti-γ-Tubulin (1:200, Sigma T6557), and rat anti-HA (1:500, Roche 12CA5). Phalloidin-TRITC (1:1000, Sigma) was used to stain F-actin.

In situ hybridization was performed as described in Lecuyer *et al.* (2007). Digoxigenin (DIG)-labeled probe was made from full-length *grk* cDNA (gift from Trudi Schüpbach). In situ signal was developed using fluorescein tyramide conjugate (gift from Sabine Reichert). X-gal staining was performed as described in Ashburner (1989).

REFERENCES

Ashburner, M., 1989 Drosophila. Cold Spring Harbor Laboratory, Cold Spring Harbor, N.Y.

Forrest, K. M., and E. R. Gavis, 2003 Live imaging of endogenous RNA reveals a diffusion and entrapment mechanism for nanos mRNA localization in Drosophila. Curr Biol 13: 1159-1168.

Lecuyer, E., H. Yoshida, N. Parthasarathy, C. Alm, T. Babak *et al.*, 2007 Global analysis of mRNA localization reveals a prominent role in organizing cellular architecture and function. Cell 131: 174-187.

Li, H., and R. Durbin, 2009 Fast and accurate short read alignment with Burrows-Wheeler transform. Bioinformatics 25: 1754-1760.

Li, H., B. Handsaker, A. Wysoker, T. Fennell, J. Ruan *et al.*, 2009 The Sequence Alignment/Map format and SAMtools. Bioinformatics 25: 2078-2079.

Misra, S., M. A. Crosby, C. J. Mungall, B. B. Matthews, K. S. Campbell *et al.*, 2002 Annotation of the Drosophila melanogaster euchromatic genome: a systematic review. Genome Biol **3**: RESEARCH0083.

Rorth, P., 1998 Gal4 in the Drosophila female germline. Mech Dev 78: 113-118.

Serano, T. L., H. K. Cheung, L. H. Frank and R. S. Cohen, 1994 P element transformation vectors for studying Drosophila melanogaster oogenesis and early embryogenesis. Gene 138: 181-186.

Shaner, N. C., R. E. Campbell, P. A. Steinbach, B. N. Giepmans, A. E. Palmer *et al.*, 2004 Improved monomeric red, orange and yellow fluorescent proteins derived from Discosoma sp. red fluorescent protein. Nat Biotechnol 22: 1567-1572.

Van Doren, M., A. L. Williamson and R. Lehmann, 1998 Regulation of zygotic gene expression in Drosophila primordial germ cells. Curr Biol 8: 243-246.

Venken, K. J., J. W. Carlson, K. L. Schulze, H. Pan, Y. He *et al.*, 2009 Versatile P[acman] BAC libraries for transgenesis studies in Drosophila melanogaster. Nat Methods 6: 431-434.

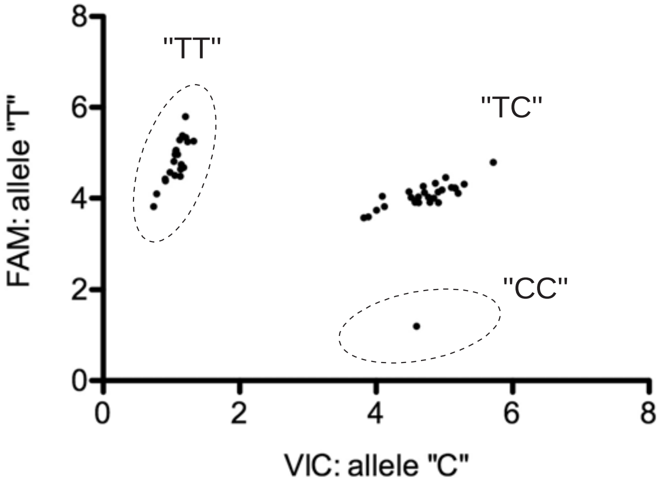

File S2 An example of KASPAr results. The results of KASPAr genotyping for SNP_75F of 48 *TM3*-bearing recombinants and a control homozygous *FRT2A* fly. KASPAr results, in arbitrary units of FAM and VIC fluorescence, reveal three distinguishable clusters corresponding to the three allelic combinations [CC (control), CT (*FRT2A/TM3*), and TT(*ru h th/TM3*)].

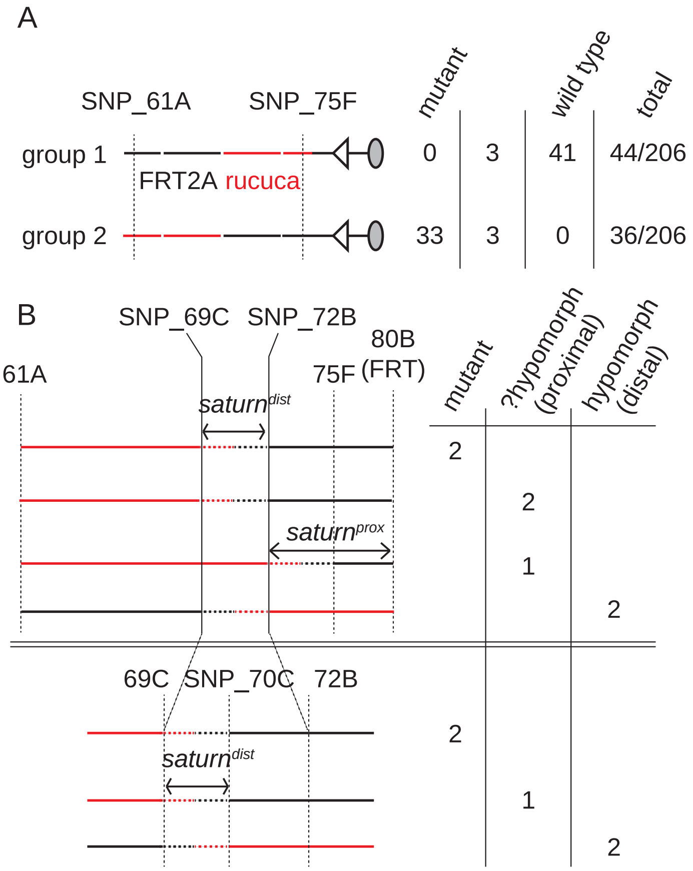

File S3 ***saturn* is caused by two mutations that are mapped to 69C-70C (*saturn*^{dist}) and 72B-80B (*saturn*^{prox}).** Summary of SNPs genotyping. (A) 206 putative recombinants were genotyped for the SNPs at 61A and at 75F. Non-recombinant lines (group 3) are excluded from the subsequent assays. 6/80 recombinant lines have a hypomorphic phenotype, suggesting that the presence of two closely-linked mutations near to 75F. (B) Initial second-stage genotyping reveals that *saturn*^{dist} lies in 69C-72B and *saturn*^{prox} mutation is between 72B and the 80B (FRT), and further genotyping places *saturn*^{dist} mutation between 69C and 70C. WGS shows that *saturn*^{prox} and *saturn*^{dist} represent mutations in *klc* and *mael*, respectively (see results).

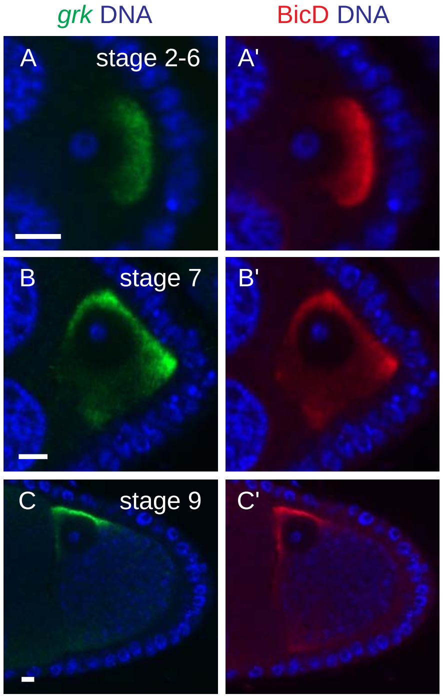

File S4 ***grk*** **mRNA colocalizes with BicD throughout oogenesis.** Co-staining of *grk*mCherry* (*grk*) and the dynein cofactor BicD in stage 2-6 (A), stage 7 (B) and stage 9 (C) wildtype egg chambers, showing that *grk* mRNA colocalizes with BicD in the oocyte throughout oogenesis. Scale bars = 5 μm

File S5

Anterior of stage 8 oocyte lack axial symmetry (movie)

3D reconstruction of confocal images of a wildtype stage 8 egg-chamber with anti-BicD staining (green) showing four nurse cells impinging on the anterior surface of the oocyte. Two of the four anteriormost nurse cells are displaced further into the oocyte and thus cause greater irregularity and deeper "corners". The positions of the oocyte nucleus and one of the nurse cell nuclei are marked. Image reconstruction was done using Imaris 7.2.

File S5 is available for download at http://www.g3journal.org/lookup/suppl/doi:10.1534/g3.114.010462/-/DC1.
