# Supplementary material for: A Genetic Screen Based on in Vivo RNA Imaging Reveals Centrosome-Independent Mechanisms for Localizing gurken Transcripts in Drosophila
Source: G3 (Bethesda). 2014 Feb 14;4(4):749–60. doi: 10.1534/g3.114.010462 (PMC4059244; doi:10.1534/g3.114.010462)
Supplement: Supporting Information [file supp_g3.114.010462_FileS2.pdf]

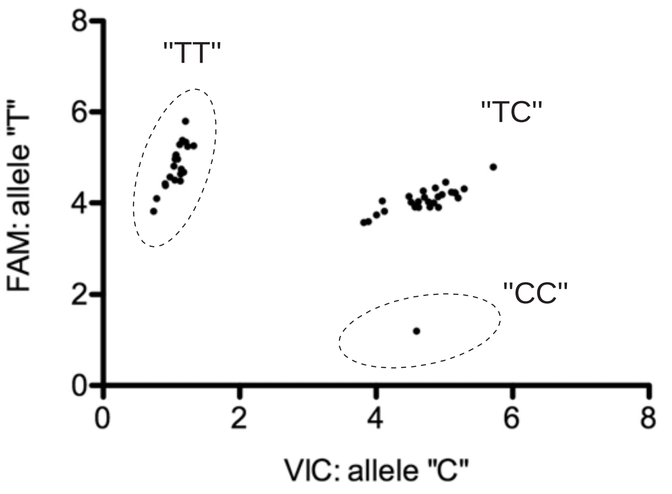

File S2 An example of KASPAr results. The results of KASPAr genotyping for SNP_75F of 48 *TM3*-bearing recombinants and a control homozygous *FRT2A* fly. KASPAr results, in arbitrary units of FAM and VIC fluorescence, reveal three distinguishable clusters corresponding to the three allelic combinations [CC (control), CT (*FRT2A/TM3*), and TT(*ru h th/TM3*)].
