# Supplementary material for: A Genetic Screen Based on in Vivo RNA Imaging Reveals Centrosome-Independent Mechanisms for Localizing gurken Transcripts in Drosophila
Source: G3 (Bethesda). 2014 Feb 14;4(4):749–60. doi: 10.1534/g3.114.010462 (PMC4059244; doi:10.1534/g3.114.010462)
Supplement: Supporting Information [file supp_g3.114.010462_FileS3.pdf]

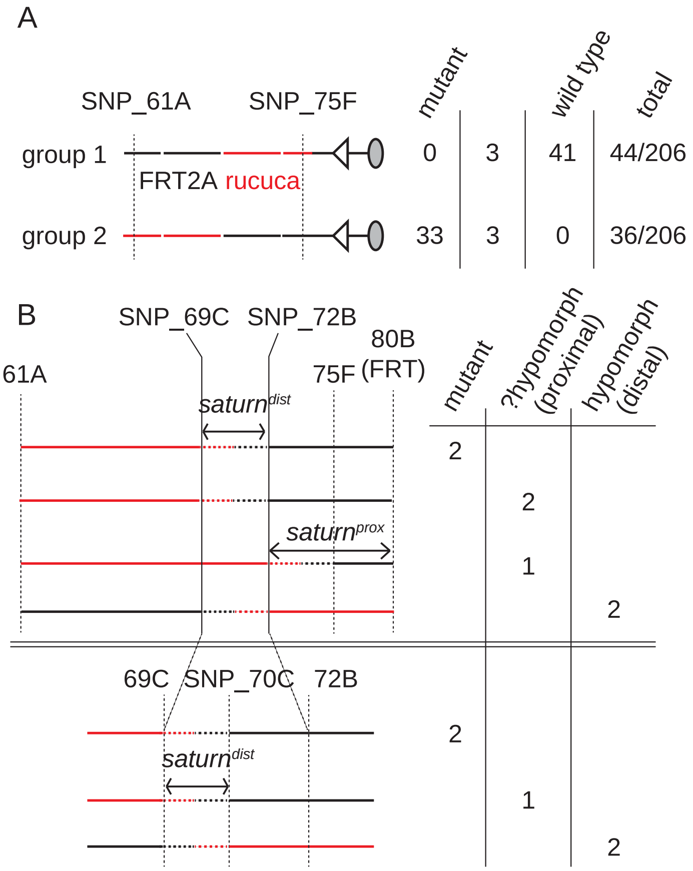

File S3 ***saturn*** **is caused by two mutations that are mapped to 69C-70C (*saturn*^{dist}) and 72B-80B (*saturn*^{prox}).** Summary of SNPs genotyping. (A) 206 putative recombinants were genotyped for the SNPs at 61A and at 75F. Non-recombinant lines (group 3) are excluded from the subsequent assays. 6/80 recombinant lines have a hypomorphic phenotype, suggesting that the presence of two closely-linked mutations near to 75F. (B) Initial second-stage genotyping reveals that *saturn*^{dist} lies in 69C-72B and *saturn*^{prox} mutation is between 72B and the 80B (FRT), and further genotyping places *saturn*^{dist} mutation between 69C and 70C. WGS shows that *saturn*^{prox} and *saturn*^{dist} represent mutations in *klc* and *mael*, respectively (see results).
