# Supplementary material for: A Genetic Screen Based on in Vivo RNA Imaging Reveals Centrosome-Independent Mechanisms for Localizing gurken Transcripts in Drosophila
Source: G3 (Bethesda). 2014 Feb 14;4(4):749–60. doi: 10.1534/g3.114.010462 (PMC4059244; doi:10.1534/g3.114.010462)
Supplement: Supporting Information [file supp_g3.114.010462_FileS4.pdf]

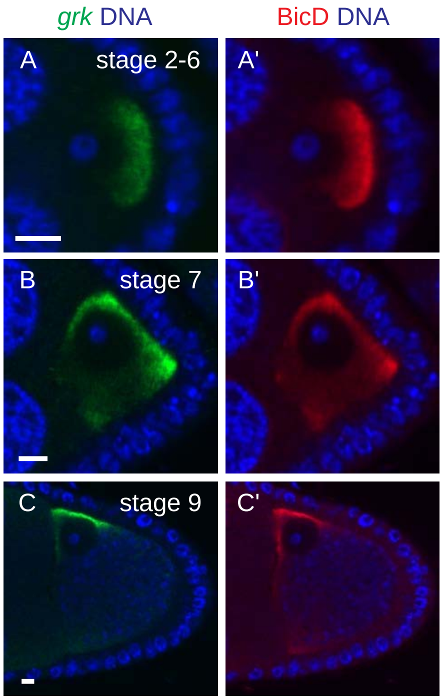

File S4 *grk* mRNA colocalizes with BicD throughout oogenesis. Co-staining of *grk*mCherry* (*grk*) and the dynein cofactor BicD in stage 2-6 (A), stage 7 (B) and stage 9 (C) wildtype egg chambers, showing that *grk* mRNA colocalizes with BicD in the oocyte throughout oogenesis. Scale bars = 5 μm
